# Extending user control for image stylization using hierarchical style transfer networks

**DOI:** 10.1016/j.heliyon.2024.e27012

**Published:** 2024-02-28

**Authors:** Sunder Ali Khowaja, Sultan Almakdi, Muhammad Ali Memon, Parus Khuwaja, Adel Sulaiman, Ali Alqahtani, Asadullah Shaikh, Abdullah Alghamdi

**Affiliations:** a Department of Telecommunication, Faculty of Eng. And Tech, University of Sindh, Jamshoro, Sindh, 76090, Pakistan; b Department of Computer Science, College of Computer Science and Information Systems, Najran University, Najran, 61441, Najran, Saudi Arabia; c Department of Information Technology, University of Sindh, Jamshoro, Sindh, 76090, Pakistan; d Institute of Business Administration, University of Sindh, Jamshoro, Sindh, 76090, Pakistan; e Scientific and Engineering Research Centre, Najran University, Najran, 61441, Najran, Saudi Arabia; f Department of Networks and Communications Engineering, College of Computer Science and Information Systems, Najran University, Najran, 61441, Najran, Saudi Arabia; g Department of Information Systems, College of Computer Science and Information Systems, Najran University, Najran, 61441, Najran, Saudi Arabia

**Keywords:** Neural style transfer, User control, Fixpoint control loss, Denoising, Hierarchical network

## Abstract

The field of neural style transfer refers to the re-rendering of content image while fusing the features of a style image. The recent studies either focus on multiple style transfer or arbitrary style transfer while using perceptual and fixpoint content losses in their respective network architectures. The aforementioned losses provide notable stylization results but lack the liberty of style control to the user. Consequently, the stylization results also compromise the preservation of details with respect to the content image. This work proposes the hierarchical style transfer network (HSTN) for the image stylization task that could provide the user with the liberty to control the degree of incurred style via denoising parameter. The HSTN incorporates the proposed fixpoint control loss that preserves details from the content image and the addition of denoising CNN network (DnCNN) and denoising loss for allowing the user to control the level of stylization. The encoder-decoder block, the DnCNN block, and the loss network block make the basic building blocks of HSTN. Extensive experiments have been carried out, and the results are compared with existing works to demonstrate the effectiveness of HSTN. The subjective user evaluation shows that the HSTN's stylization represents the best fusion of style and generates unique stylization results while preserving the content image details, which is evident by acquiring 12% better results than the second-best performing method. It has also been observed that the proposed work is amongst the studies that achieve the best trade-off regarding content and style classification scores, i.e. 37.64% and 60.27%, respectively.

## 1 Introduction

Style transfer is a process to synthesize or reconstruct the texture of a content image with reference to the target style. In this regard, the style transfer is quite similar to the texture synthesis, where both techniques use certain constraints to model the statistics of the content or the style image. The constraints in texture synthesis focus on smoothing the boundary transitions between two images. In style transfer, the constraints focus on preserving the structural details in the content image. Some studies also regard style transfer as a generalized form of texture synthesis [1]. The existing studies employing texture synthesis were based on filterbanks [2], multi-scale feature statistics [3], [4], and pixel resampling [5], [6]. Since the inception of convolutional neural networks (CNN), the style transfer and texture synthesis problems have been revisited by using pre-trained discriminative learning architectures as a feature extraction mechanism [7]. Recently, transformer networks with attention mechanism have been gaining significant interest due to their style mixing performance [8]. Gatys et al. [7] used a pre-trained visual geometry group (VGG) [9] network to extract feature correlations for migrating the style to the content image. The framework revived the interest in style transfer and texture synthesis by either training feed-forward networks [10], [11], [12], [13] or using iterative optimization techniques [7], [14], [15]. The deep architectures allow the researchers to extract rich feature representations that yield better visual results than traditional texture synthesis methods. Some existing works encode the feature maps, i.e., mean and variance, in 1-D space, which is difficult to represent. Existing works suggest that the feature representations from Gram Matrix yield better visual results in comparison to the optimization-based 1-D style representations [16], [17]. The 1-D encoding of feature maps potentially limits the flexibility of feature map extraction and exploration of style representations. In addition to the visual quality, concerns over the flexibility of representation have also been raised in [18], which affects the brush stroke size and the control of style transfer. The changes in the brush stroke size naturally occur when the style image is resized to match the content image. The change in image size also affects the mapping of texton (texture element), which is convolved with the Delta function to transfer style to the content image. The Delta function in image space is highly related to the sampling positions. The simple convolution of texton and Deltas in the original image space introduces boundary discontinuity problems, thus leading to artefacts. Recent works have used downsampling and upsampling at the encoder and decoder levels to overcome the issues of brush stroke size [19], explicit representation, and content/style separation, respectively. On the other hand, the control of style transfer is not given much attention. The problem of brush stroke size is also indirectly related to the style transfer control, such as image composition (sky and ground), fine spatial structures (texture and shape), coarse-scale structures (swirly effects in the painting), and colour palette [7]. We also add the blurring or cartoonish effect (where artistic effects overshadow the fine details) to the problem of style transfer control. Gatys et al. in [20] introduced the control problem in style transfer regarding colour palettes and spatial structures. Yang et al. [21] dealt with the transfer control specifically for textual images, and Liu et al. [22] represented the preservation of salient regions as a control problem in neural style transfer. This work focuses on the style transfer control problem related to the blur and cartoon effect and the spatial control for preserving fine and coarse spatial structures.

Some of the existing studies have used generative adversarial networks (GANs) along with attention mechanisms to improve upon the stylization aspect [23], [24], [25], [8]. Although the performance in terms of visualization and preservation of certain image aspects is improved, depending on the losses and aim of the desired study, such networks have two major drawbacks. The first is the training computational complexity, which is higher relative to the CNN-based encoder-decoder networks [8], [26], and the second is the lack of stylization control with the transformer networks, unless and until it is explicitly trained on varying a certain parameter through the extrinsic network or style path [8], [27]. The former problem limits the training of stylization networks on CPUs while the latter lacks control. Therefore, stylization networks that can cater to both the problems are in dire need.

In this work, we propose a hierarchical style transfer network (HSTN) that not only is able to be trained on CPUs but also provides a degree of style control to the users. The HSTN extracts the feature maps using an encoder-decoder network for transferring style to the content image. The network produces an explicit feature representation which will be an input to a loss network along with the original content and style image. We propose fixpoint control loss and denoising loss to control the style transfer accordingly. Furthermore, the HSTN network will also allow transferring the styles from more than one image by switching the style image at the hierarchical step, i.e., the style input to the loss network. The HSTN can also be used for incremental learning by holding the encoder-decoder network fixed and training the loss network with different styles and content images since there is no need for training from scratch for multiple styles. Furthermore, the HSTN will provide more opportunities to create new fusion styles. For instance, the encoder-decoder network uses Van Gogh's painting, whereas the loss network uses Picasso's work. The contributions of this work can be summarized below:

We propose a hierarchical style transfer network (HSTN) for neural style transfer. We propose a fixpoint control loss and the use of denoising loss for HSTN. The hierarchical nature of the network provides flexibility for the fusion of multiple styles and for creating a new style image. Extensive analysis has been carried out to compare the proposed work with the existing ones.

The rest of the paper is structured as follows. We consolidate the related works in Section 2. Section 3 details the proposed HSTN network's technical design and the computations. Section 4 discusses the feature maps from different pre-trained networks and their impact on style transfer. The experimental results and comparative analysis are provided in Section 5. We present the conclusion and future works in Section 6.

## 2 Related work

Neural style transfer uses non-parametric sampling of patches [28], [29] or pixels [5] to grow textures, which is quite similar to texture synthesis. More appropriately, neural style transfer can be referred to as texture transfer [30], [31] problem, which synthesizes the contents from the source image while constraining the texture of the style image. The work in [32] proposed the concept of image analogies, which extracts the low-level image features from the already stylized image and transfers the texture onto the target image. Most of the texture transfer studies focused on representing and extracting semantic image content to transfer the style to the source image. However, back then, the task of separating style from the content images was still a difficult problem but with the advent of discriminative learning methods (deep CNN architectures), the problem of representing and extracting style is better mitigated [33].

One of the earliest attempts in generating artwork was the DeepDream [34], which used CNNs as its base architecture. Inspired by DeepDream, Gatys et al. [7] proposed using pre-trained VGG-16 architecture to find the correlations between style, content, and additive white Gaussian noise (AWGN) generated image for performing the style transfer. The stylized images obtained were eye-catching, with better visualization than existing texture transfer studies. Li and Wand [14] extended the work of Gatys et al. The study in [7] integrated the CNNs with Markov Random Fields (MRF) to perform patch-based and portrait painting style transfer [35]. Liao et al. [15] proposed a deep image analogy technique to solve the neural style transfer, referred to as the visual attribute transfer problem. The aforementioned studies for style transfer were based on iterative optimization methods, which were computationally expensive for real applications. Moreover, these studies did not address the transfer control problem.

An alternative to iterative optimization methods was generator networks that could provide the stylized results with a forward pass, making the generator networks run-time efficient [7]. Ulyanov et al. [12] proposed lightweight feed-forward network architecture for style transfer and texture synthesis. Johnson et al. [10] proposed the use of perceptual loss with an iterative optimization method to improve the real-time efficiency of image style transfer. Similarly, Li and Wand [11] in continuation to their previous work, proposed a Markovian General Adversarial Network (GAN) to speed up the process of style transfer. Although the real-time efficiency was improved with the use of generator networks, the learned architectures were only trained to deal with a single specific style. Thus, to add more styles the whole architecture needed to be retrained, which limited the flexibility and scalability of style transfer applications.

Following works [3], [36], [37], [17], [38], [39], [40], [19] were proposed recently to deal with arbitrary and multiple style transfers. Dumoulin et al. [37] proposed an arbitrary style of deep architecture that projects a painting in an embedding space as a single point. These points representing individual paintings are then combined arbitrarily to explore new styles. Li et al. [38] presented a single generative feed-forward network capable of synthesizing multiple styles efficiently. The work in [19] proposed a multi-style generative network that uses a Co-Match layer to extract second-order feature statistics from the content image and match corresponding features to the target styles. Study [36] used an optimization objective with a layer of the pre-trained network to match the style textures and content image structures for arbitrary image style transfer. The work in [17] proposed an adaptive instance normalization layer to align the mean and variance from style and content features for allowing multiple style transfers. This work also dealt with spatial and colour transfer control. In [39], the authors proposed using whitening and colouring as the pair of transforms to match the feature covariance of style and content image. The feature embedding from the covariance matching allows transferring multiple styles to the content image. The above-mentioned studies either represent multiple style transfers with different scaling/shifting parameters of the normalization layer [37] or with a one-hot vector encoding scheme [38]. For the arbitrary style transfer, the studies extract the feature statistics from the style/content image while using a pre-trained network architecture, say VGG-16, and retrain an encoder-decoder style network to project the extracted features onto the image space. The methods produce visually good results in terms of stylization. However, the representation of styles in their networks is not explicit. Moreover, the studies do not consider the style transfer control in terms of blur and cartoon effect.

Recently, methods have been proposed to improve the stylization and transfer of multiple styles within a single network architecture. The work in [40] proposed using StyleBank, which uses an autoencoder to transfer the style image into feature embedding space. Study [41] proposed a multi-stroke style transfer network based on a self-attention mechanism. In [42], the authors based their method on the fact that artists like to try different styles. Therefore, they proposed using disentanglement loss and fixpoint triplet loss to transfer multiple styles. There are quite a few works that solely focus on style transfer control, such as [20] focused on spatial, colour, and scale control from the style images using perceptual factors. Study [21] proposed using an adjustable parameter to control the stylistic degree in textual images. The work in [22] proposed using region loss to preserve the details from salient regions of the content image. In [43], the authors proposed using convolution-long-short term memory (Conv-LSTM) modules and multiple instance normalization layers to incorporate different styles and maintain temporal consistency for neural style transfer tasks. Study [44] raised concerns over feature extraction techniques, weights and biases of pre-trained network employed, network depth, and training procedure, accordingly. The study conducts several ablation studies and provides recommendations based on the obtained results. One highlighted recommendation was using a CNN with heavily patterned weights and fewer parameters. In [45], there was a focus on facial-based neural style transfer by applying segmentation before the style transfer. One of the highlights here is preserving the facial features while transferring the texture to the content image.

The study in [46] proposed the StyleTune network that focused on the stroke adjustments on local as well as global parameter levels. Furthermore, the StyleTune network also integrated the image super-resolution method to increase the output fidelity of the image. Jing et al. [47] presented the idea of modelling semantic correspondences of many style images into one image using semi-parametric graph neural networks. Their method models local patch correlations with attention mechanism and content and style nodes in a learnable strategy while controlling the number of edges at the inference stage, thus, reducing the neural style transfer time. Batziou et al. [23], instead of modelling multiple style transfers opted for improving the results for a specific artistic style transfer using a combination of adaptive bidimensional empirical mode decomposition and CycleGANs. The correspondences for specific styles were modelled using bidimensional intrinsic mode functions. Kong et al. [24] focused on the misaligned representations generated from network architectures for style transfer networks. In this regard, they proposed the similarity loss for embedded features and multichannel correlations to improve the representation alignment, thus improving the quality of style transfer images. An et al. [48] opted for a different approach, i.e. evaluating different network architectures for style transfer to provide a quantitative analysis for choosing the most efficient one. It was suggested that adding a deep feature alignment strategy to VGG19 or any other architecture would improve the computational efficiency of the network and style transfer output. Similarly, Ma et al. [25] proposed restorable arbitrary style transfer networks that focused more on restoring the style transfer images rather than explicitly improving the transfer mechanism. The work proposed style difference and multi-restoration losses to preserve content and style embedding.

Our work leverages the characteristics of StyleBank [16] and different control losses to explicitly integrate multiple styles and control the style transfer. However, unlike StyleBank, we do not use StyleBank layers. Rather, we explicitly input the extracted feature maps to HSTN for integrating multiple styles. Furthermore, instead of explicitly making the CNN heavily patterned [44] and pre-processing to preserve details [39], the proposed HSTN uses the fixpoint control loss and denoising loss in its encoder-decoder block and loss network block to achieve better stylization while retaining the content image details.

We comprehensively compare existing works with the proposed one in Table 1. The comparison is performed with respect to different characteristics such as multiple style integration, consideration of style control, consideration of content preservation, and integration of cartoon effect within the learnable network architecture to highlight the novelty and uniqueness of HSTN.

**Table 1:** HSTN comparison with existing terms regarding multiple style integration, style control, content preservation, colour and transfer control, and cartoon effect integration, respectively.

Study	Year	Multiple-styles	Style control	Content Preservation	Colour and Transfer Control	Cartoon Effect
Gatys et al. [49]	2016	✗	✗	✗	✗	✗
Domoulin et al. [37]	2017	✓	✗	✗	✗	✗
Huang and Belongie [17]	2017	✓	✗	✗	✓	✗
Li et al. [38]	2017	✓	✗	✗	✓	✗
Gatys et al. [20]	2017	✗	✓	✗	✗	✗
Zhang and Dana [19]	2019	✓	✗	✗	✗	✗
Yang et al. [50]	2019	✓	✓	✗	✗	✗
Liu et al. [22]	2019	✓	✗	✓	✗	✗
Chen et al. [40]	2020	✓	✗	✗	✓	✗
Liu et al. [51]	2021	✓	✓	✗	✗	✗
Reimann et al. [46]	2022	✓	✓	✓	✗	✗
Jing et al. [47]	2023	✓	✓	✓	✗	✗
Batziou et al. [23]	2023	✗	✓	✓	✗	✗
Kong et al. [24]	2023	✓	✓	✓	✗	✗
Ma et al. [25]	2023	✓	✗	✓	✓	✗
Ours	2023	✓	✓	✓	✓	✓

## 3 Hierarchical style transfer networks

**Figure 1:**
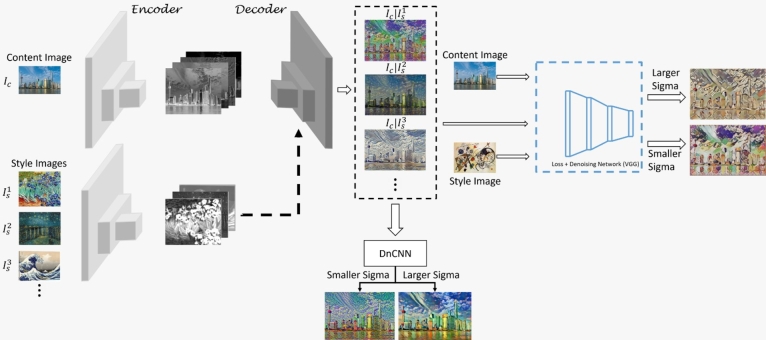
An overview of the proposed hierarchical style transfer network.

The proposed hierarchical style transfer network (HSTN) is shown in Fig. 1. It can be noticed that the HSTN comprises three blocks. The first is the encoder-decoder style network that creates multiple fusions of styles using a series of losses, which helps in style fusion and control. The second block is the denoising convolutional neural network (DnCNN) that enhances the art of the stylized image with respect to the sigma value. The lower sigma value retains the fine details which in turn reduces the amount of style infused whereas the higher sigma value enhances the style information and adds a cartoonish effect, respectively. The third and final is the loss network block that uses the output from the first block as an additional style and embeds the style fusion to images that the network has not seen before. The details for each of the blocks are provided in the subsequent subsections. Table 2 represents nomenclature with variable and its description.

**Table 2:** Nomenclature table.

Variable	Description
*EC*	Encoder for the content image
*DC*	Content decoder for the content image
*ES*	Network generates a vector from style images
*p*	Content Image
*q*	Style Image
*θ*	Trainable parameter
*F*(*I*_*o*_)	output residual map
*I*	number of training image pairs
*Loss*_*pp*	Perceptual loss
*Loss*_*ct*	Content Loss
*Loss*_*st*	Style Loss
*Loss*_*vr*	Variation regularization loss
*Loss*_*fr*	feature reconstruction Loss
*Loss*_*dn*	denoising loss
$I_{C}^{\prime }$, $I_{S}^{\prime }$, $I_{O}^{\prime }$	content, style, and output image
*ρ*, *ω*, *ϕ*, *σ*, and *ψ*	Are the corresponding weights for content
*ς*, and ℑ	Gram matrix and feature maps

### 3.1 Encoder-decoder block

The encoder-decoder architectures have been extensively used for image-to-image translation using supervised and unsupervised techniques, respectively [52], [53]. In this work, we also use encoder-decoder network architecture such that the encoder for the content image is denoted as ES representing EC, the encoder for the style image, and the content decoder as DC, accordingly. The EC and DC are fully feed-forward convolution neural networks for generating images, whereas the ES network generates a vector from style images $ES(I_{s})$. Such type of style networks mainly relies on the minimization of losses specific to the defined tasks. One of the reasons for proposing HSTN is the style control by preserving fine details. Therefore, we use the reconstruction loss that enforces the style transformation to resemble the content image. The reconstruction loss is given in Equation (1).

(1) $${\mathrm{Loss}}_{\text{rec }}:=\underset{\begin{array}{c} p∼I_{c}\\ q∼I_{s} \end{array}}{E_{p}}\left[{\left\|(DC(ES(q),EC(p)))-p\right\|}_{2}^{2}\right]$$

where p is the content image, and q is the style image. The reconstruction loss ensures the dominancy of the content image. However, using only reconstruction loss will lead to pixel-level similarity. Thus, the integration of style will be obstructed. In literature, a fixpoint content loss [42] has been used to let the encoder for content images decide what features must be preserved during image stylization. The fixpoint content loss is shown in Equation (2).

(2) $${\mathrm{Loss}}_{fp}:=\underset{\begin{array}{c} p∼I_{c}\\ q∼I_{s} \end{array}}{E}\left[{\left\|EC(DC(ES(q),EC(p)))-EC(p)\right\|}_{2}^{2}\right]$$

Studies have shown that the fixpoint content loss is effective, considering that the style should be infused with one image. However, the loss is ineffective when dealing with more than one fused style image [42]. Furthermore, using the aforementioned two losses unintentionally conditions the style of the content image. Another problem in terms of multiple style transfer occurs such that the visual presentations of two different styles appear to be identical when mapped onto the same point, i.e., $DC(EC(I_{c}),ES(I_{s}^{1}))$ ≡ $DC(EC(I_{c}),ES(I_{s}^{2}))$. The intuition for proposing the fixpoint control loss is to add a constraint to the stylization process such that it is bounded to the input style image while preserving the fine details of the content image. The loss undertakes the positive and negative samples concerning the anchor to ensure that the intuition behind fixpoint control loss is realized within the training process. To overcome the aforementioned issues, we propose the fixpoint control loss. The reason for proposing the said loss is three-fold. The first is related to the enforced stylization with respect to the input style image. The second is to make the stylization from two different style images distant regarding representation space. The third is to make the content image dominant and enforce its resemblance. The proposed fixpoint control loss is given in Equation (3).

(3) $${\mathrm{Loss}}_{fpc}:=E\underset{\begin{array}{c} p∼I_{c}\\ (q1),(q2)∼I_{s} \end{array}}{}\max(0,{\left\|(DC(ES(q),EC(p)))-p\right\|}_{2}+{\left\|ES(q1)-ES(DC(EC(p),ES(q1)))\right\|}^{2}-{\left\|ES(q1)-ES(DC(EC(p),ES(q2)))\right\|}^{2})$$

In equation (3), the first term is for the resemblance enforcement of the content image, the second term is the positive sample, and the third term is the negative sample with respect to the anchor ES(q1). We can use the fixpoint control loss for N-style images to generate different styles as shown in Fig. 1.

### 3.2 DnCNN block

To the best of our knowledge, no other study uses a denoising network to enhance the stylization of a content image. This study uses DnCNN [54], [55], [56] to add a cartoonish effect to the stylized image. To achieve this, we fine-tune the pre-trained DnCNN on the CBSD [57] dataset while generating their cartooned version on several scales and associating them with the sigma level. In this study, we opt for the sigma values 10 and 25 and refer to them as smaller and larger sigma values, respectively. The advantage of using DnCNN is that the output preserves the content image details while smoothing the features from the style image. Furthermore, the basis of this study is to control the style level, which is achieved by varying the sigma level, accordingly. The denoising loss used for the DnCNN block is given in Equation (4).

(4) $${\mathrm{Loss}}_{dn}(\vartheta )=\frac{1}{2N}\sum_{i=1}^{I}{\left\|F\left(I_{O};\vartheta \right)-\left(I_{O_{i}}-I_{D_{i}}\right)\right\|}_{F}^{2}$$

where *ϑ* is the trainable parameter, $F(I_{O})$ is the output residual map from denoising block, $I_{O}=[I_{C}|I_{S}^{1},I_{C}|I_{S}^{2},...$ and $I_{D}=I_{O}-F(I_{O})$, accordingly. The DnCNN is fine-tuned to generate the $F(I_{O})$, which is then subtracted from the output of the decoder block $I_{O}$. We add zero-mean white Gaussian Noise and varying sigma level to the images in CBSD dataset. To fine-tune the DnCNN network, we used stochastic gradient descent (SGD) optimizer by initiating the learning rate to 0.001 and a decay rate of 0.01. The DnCNN network was trained for 35 epochs with a batch size of 64 and default settings for other hyperparameters, respectively. The fine-tuning of the DnCNN block is in compliance with the study [58]. The notation *I* and $I_{D}$ refer to the number of training image pairs, i.e., $\left(I_{O},F(I_{O})\right)$, and the output from image denoising network, respectively. For generating the cartooned version of the output stylized images on various scales, we used the method proposed in [54].

### 3.3 Loss network block

Once the image is stylized using the Encoder-decoder block and the denoising block, the user has two options: they can either get the stylized image using the available style images or provide their own style image to blend in. For the loss network block, we use well-known perceptual loss ($Loss_{pp}$) [10], which comprises content ($Loss_{ct}$), style ($Loss_{st}$), and variation regularization loss ($Loss_{vr}$) [55], along with the feature reconstruction ($Loss_{fr}$) [10] and denoising loss ($Loss_{dn}$) [56].

The formulation for the perceptual loss is given in Equation (5).

(5) $${\mathrm{Loss}}_{pp}\;\left(I_{C}^{\prime },I_{S}^{\prime },I_{O}^{\prime },\vartheta \right)=\rho {\mathrm{Loss}}_{ct}\;\left(I_{O}^{\prime },I_{C}^{\prime }\right)+\omega {\mathrm{Loss}}_{st}\;\left(I_{O}^{\prime },I_{S}^{\prime }\right)+\varphi {\mathrm{Loss}}_{vr}\;\left(I_{O}^{\prime }\right)+\sigma {\mathrm{Loss}}_{dn}(\vartheta )+\psi {\mathrm{Loss}}_{fr}\;\left(I_{O}^{\prime },I_{O}\right)$$

where $\left(I_{C}^{\prime },I_{S}^{\prime },I_{O}^{\prime }\right)$ are the content, style, and output image for the hierarchical step, i.e., loss network block. The variables ρ,ω,ϕ,σ and *ψ* are the corresponding weights for content, style, variation regularization, denoising, and feature reconstruction loss, respectively. We use the pre-trained VGG16 network [33] for training the loss network. The style, content, and feature reconstruction losses are defined in Equations (6), (7), and (8), accordingly.

(6) $${\mathrm{Loss}}_{st}\;\left(I_{o}^{\prime },I_{s}^{\prime }\right)=\sum_{l\in \left\{l_{st}\right\}}{\left\|\varsigma \left({\overset{˜}{ℑ}}^{l}\left(I_{o}^{\prime }\right)\right)-\varsigma \left({\overset{˜}{ℑ}}^{l}\left(I_{s}^{\prime }\right)\right)\right\|}^{2}$$

(7) $${\mathrm{Loss}}_{ct}\;\left(I_{O}^{\prime },I_{c}^{\prime }\right)=\sum_{l\in \left\{l_{ct}\right\}}{\left\|{ℑ}^{1}\left(I_{O}^{\prime }\right)-{ℑ}^{1}\left(I_{c}^{\prime }\right)\right\|}^{2}$$

(8) $${\mathrm{Loss}}_{fr}\;\left(I_{O}^{\prime },I_{O}\right)=\sum_{l\in \left\{l_{fr}\right\}}{\left\|{ℑ}^{l}\left(I_{O}^{\prime }\right)-{ℑ}^{l}\left(I_{O}\right)\right\|}^{2}$$

where *ς* and ${ℑ}^{l}$ refer to the Gram matrix and feature maps obtained from VGG-16 pre-trained network layer *l*. The subscripts for the *l* represent the functionality of the layer with respect to the loss function. We used the same computation of variation regularization loss as suggested in [10].

### 3.4 Network architecture for encoder-decoder

We adopted the network architecture suggested in [16], [10]. The encoder network comprises 3 convolutional layers, the first convolutional layer has a stride of 1 with 32 channels while the remaining 2 convolutional layers have 2 strides with a number of features set to 64 and 128, respectively. The decoder part has half fractionally strided- two stride and one stride-1 convolutional layer, accordingly.

The instance normalization [59] and rectified linear unit (ReLU) activation function were applied after each convolutional layer, except the last one. Multiple studies have proved that the instance normalization layer performs better for dealing with background artefacts introduced due to padding compared to the spatial batch normalization layer [16], [40], [60]. The first and the last convolutional layer have a kernel size of 7x7, while the remaining layers use a 3x3 kernel size. In contrast to the network proposed in [10], we eliminate the residual blocks to reduce the computational overhead. The aforementioned configuration is also shown in Table 3.

**Table 3:** Details of network configuration for encoder-decoder network.

Layer	Stride	Kernel size	Number of Channels
Conv1	1	7x7	32
Conv2	2	3x3	64
Conv3	2	3x3	128
Deconv1	2	3x3	128
Deconv2	2	3x3	64
Conv4	1	7x7	3

### 3.5 Network training

The Encoder-Decoder network is trained using randomly sampled images from the Microsoft COCO dataset [61] amongst 1000 content images while the style images are borrowed from various research articles and the Internet. The content image is cropped to 512x512 while the style image is scaled to 500. The network was trained for 400k iterations using a batch size of 5. The Adam optimizer [62] was used with an initial learning rate of 0.025 with a decay rate of 0.3 at every 10k iterations.

We use the same COCO dataset for the Loss network; however, we resize the content image to 256x256 while scaling the style image to 200 on the long side. The network was trained for 50k iterations with a batch size of 4. The Adam optimizer was used with an initial learning rate of 0.0045 and a decay rate of 0.1 after every 5k iterations. We compute the feature reconstruction and content loss at relu2_2, style loss at relu1_2, relu2_2, relu3_2, and relu4_2, and the denoising loss at relu4_3 of the pre-trained VGG16 network. The method has been implemented and tested on a PC with 32GB RAM, corei5 clocked at 3.4 GHz, and a GeForce GTX 1080Ti GPU using MATLAB. The training roughly takes around 12 hours on the aforementioned configuration

## 4 Experimental results

The study's main goal is to propose a hierarchical style transfer network that can generate unique multiple styles while controlling the style transfer in an autonomous manner rather than achieving state-of-the-art results. The control of style transfer includes the cartoonish effect as well. However, following the convention, we compare our results qualitatively to other style transfer approaches based on optimization methods [7], single-style methods [10], [12], multiple-style methods [40], [37], [38], [19], and arbitrary style methods [17], [63], accordingly. The results for comparison are either borrowed from existing papers or using the open-source codes from the authors. We denote the results obtained using the second block of the HSTN as HSTN-D and the results obtained from the loss network as HSTN-L, respectively.

### 4.1 Comparison with optimization-based methods

**Figure 2:**
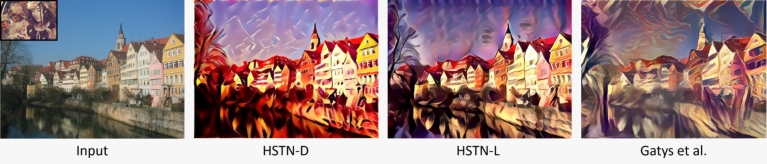
Comparison of HSTN with iterative optimization method [7].

The iterative optimization-based methods [7] used a combination of losses such as content, style, and total variation loss to mimic the perceptual stylization. The results obtained using HSTN-D, HSTN-L, and [7] are compared qualitatively in Fig. 2. It can be noticed that the feed-forward network in [7] creates larger abstract stylization, whereas the HSTN-D adds a cartoonish effect and HSTN-L generates less stylized abstraction but preserves some details such as tree and river textures. It is subjective to judge which qualitative results are better but in terms of computational complexity, the HSTN is a lot faster than the optimization-based methods, which are compliant with many feed-forward network-based studies.

### 4.2 Comparison with single-style methods

**Figure 3:**
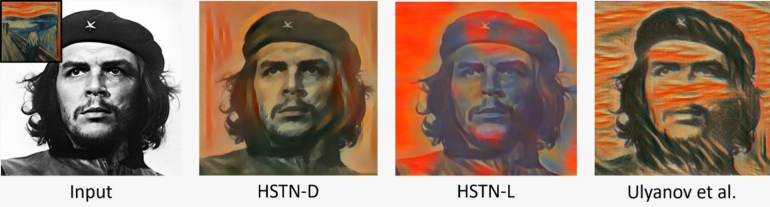
Comparison of HSTN results with the single style method proposed in [12].

**Figure 4:**
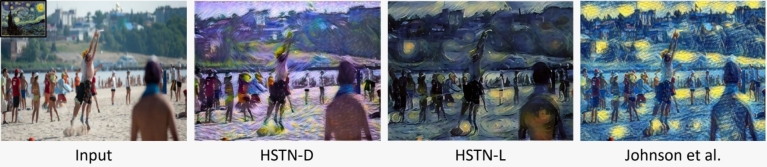
Comparison of HSTN with the single style method proposed in [10].

We compare the results of HSTN-D and HSTN-L with the studies proposed in [10], [12] in Figure 3, Figure 4, respectively. The study [12] considers the style transfer problem as the task of synthesizing texture using a shallow learning technique. It can be noticed from the said work that the texture is randomly pasted onto the image for the sake of stylization. The work in [10] uses a deeper network but produces a deep dream stylization. In contrast, the proposed HSTN depicts a style transfer based on regions visualized from details preservation.

### 4.3 Comparison with multiple-stylization methods

The researchers have now moved to arbitrary stylization and multiple-stylization methods. The former uses first and second-order statistics to extract style features for transforming the content features, followed by the training of a decoder that can generate the stylization results by transforming the content features in the image space. The latter often uses one-dimensional parameter scaling/shifting with respect to the instance normalization layer or uses a one-hot vector encoding scheme to represent different styles. In this subsection, we would compare the HSTN's stylization results with the above-mentioned stylization methods proposed in [40], [37], [17], [38], [63], [19].

**Figure 5:**
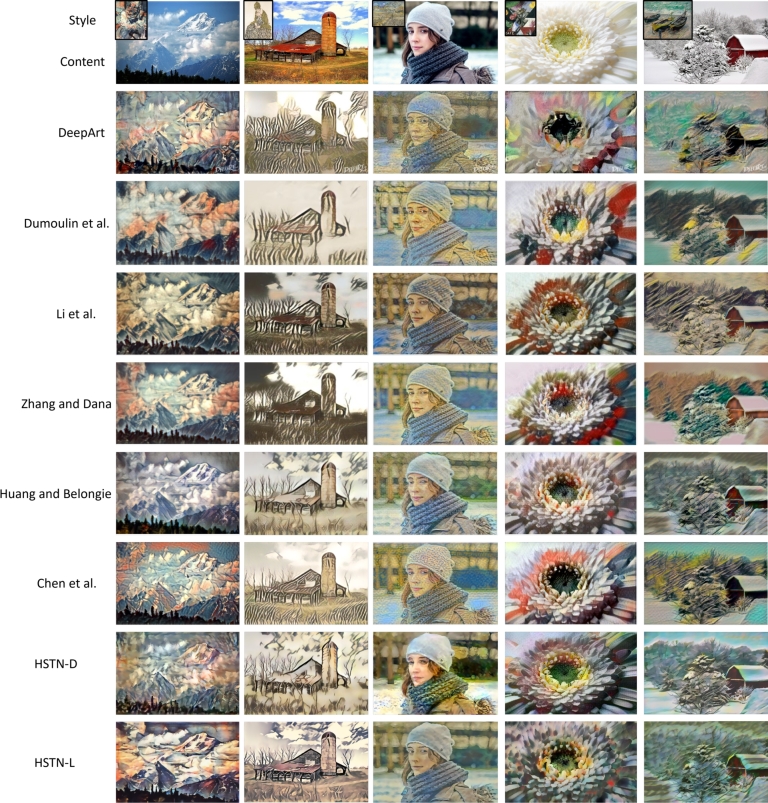
Qualitative comparison of different stylization results with HSTN.

The qualitative comparison is shown in Fig. 5. We either extract the stylization results directly from the said paper or use their implementation and demos available online. The results show that the multiple-stylization method leverages different style elements for learning to stylize a specific image. On the other hand, arbitrary style methods yield broken style elements and distorted content structures with blurry effects, but in their defence, they can handle arbitrary styles. Our method also yields blurry effects but adds cartoonish effects while preserving the details that can be visualized from the results. With our subjective interpretation, we believe that the work by Zhang and Dana [19] generates some unique stylization, while Chen et al. [40] is more focused on region-based stylization. Dumoulin et al. [37] work is more visually appealing. However, the proposed work amalgamates region-based stylization and style control; therefore, some unique and visually appealing results are obtained using HSTN. Subsequently, we also performed a subjective quantitative analysis on the stylization results obtained using HSTN and existing works. Our evaluation provides 30 users with 75 stylization results (43% females and 57% males). The age-wise proportion of the users is 50% users (15-25), 26.7% (26-35), 16.7% (36-45), and 6.6% (46-55), accordingly. The field study or specialization proportion of the users is 63.3% (Engineering), 10% (Social Sciences), 10% (Natural Sciences), and 16.7% (Arts), accordingly.

We asked the following questions to the users:

1. Select the method representing the best blend of style.
2. Select the method representing the best preservation of details with respect to the content image.
3. Select the method representing a unique stylization.
4. Select the method with the results most visually striking.

The results from the user responses are presented in Table 4. It can be noticed that the HSTN consistently acquires better user evaluation on the above-mentioned questions. It should also be considered that the user evaluation was subjective, and many users did not know about the technicalities of the methodology or neural style transfer in general. Therefore, uncertainties may exist when it comes to the method comparison. It was also noticed that the users who evaluated the results were more inclined to the colourful and dark style elements. In contrast, the stylization with more exposure and brightness got less response even though they represented the correct stylization. Such evaluation is consistent with many studies as the quantitative evaluation only focuses on the similarity aspect and does not account for the stylization part. Meanwhile, stylization corresponds to the user's subjectivity and particular interest. Recent studies such as DualStyleGAN [27], Batziou et al. [23], Kong et al. [24], and Ma et al. [25], adopted similar form of evaluation, respectively. The results show that the proposed work yields better results than the existing approaches in preserving details and the visually striking, unique stylization, and best blending, respectively.

**Table 4:** User study evaluation for different stylization methods.

Method	a.	b.	c.	d.
(Dumoulin et al., 2017) [37]	19.1%	10.0%	10.8%	10.7%
(Y. Li et al., 2017a) [38]	17.5%	15.0%	11.7%	14.0%
(H. Zhang & Dana, 2019) [19]	13.3%	10.0%	11.7%	18.2%
(Mao et al., 2017) [63]	7.5%	11.7%	15.8%	6.5%
(Huang & Belongie, 2017) [17]	8.3%	10.8%	12.5%	9.0%
Chen et al. (D. Chen et al., 2020) [40]	10.0%	12.5%	13.3%	13.2%
HSTN	24.3%	30.0%	24.2%	28.4%

### 4.4 Quantitative analysis

We train our network for style transfer on MS-COCO [61] and WikiAr [64] datasets to perform quantitative analysis. The images are randomly cropped to the 256-sized square image. We perform the comparison on various state-of-the-art approaches such as multichannel correlation network (MCCNet) [24], style transfer using transformers (Stytr2) [65], adaptive attention normalization (AdaAttN) [51], ArtFlow an unbiased framework [66], multi-adaptation style transfer (MAST) [67], style attention networks (SANet) [50], attention-aware multistroke style transfer (AAMS) [41], whitening and colouring transform (WCT) [39], adaptive instance normalization (AdaIN) [17], and neural style transfer (NST) [49], respectively. The quantitative analysis uses style and content classification models to assess the performance of HSTN and other state-of-the-art approaches. The classification score undertakes the style migration and the content structure accordingly. In this regard, we use the denoising loss with a smaller sigma value, i.e., 10. Several stylized images were generated using the aforementioned methods and HSTN. The stylized images are then fed to the content and style classification models. High accuracy for content classification refers to the ability of the method to preserve the details concerning content images. In contrast, the high accuracy for style classification characterizes the ability of the method to learn representations and information concerning effective style transfer. The studies achieving higher style classification scores correspond to the fact that they do not preserve the details from the content image. On the contrary, the higher classification score for content suggests that the learned representations limit the stylization transference. The results of the quantitative analysis are reported in Table 5. In addition, the results also showcase the effect of classification scores concerning content and style when the sigma value is varied. With the larger sigma value, the cartoon effect gets more and more dominant, thus the preservation of the fine details with respect to content image is compromised. Where the classification scores for style improve significantly with increasing value of *σ*, the content classification score decreases. We believe that the blur effect is the main reason for the decrease in content classification score. An interesting effect we noticed while varying sigma value was the increase in inference time as with sigma=75, the inference time increases by 0.07 seconds, accordingly. These results support our claim regarding the user control for image stylization such that the higher sigma value yields more stylized image while the lower sigma values preserve the content image fine details.

**Table 5:** Comparative analysis of HSTN with state-of-the-art approaches using style and content classification scores.

Method	Content Classification Score	Style Classification Score	Inference time (seconds/image)
NST [49]	31.79	54.53	37.211
AdaIN [17]	39.34	56.42	0.008
WCT [39]	21.38	62.17	0.579
AAMS [41]	37.82	56.65	2.173
SANet [50]	27.14	66.49	0.019
MAST [67]	26.91	57.04	0.096
ArtFlow [66]	36.68	41.82	0.664
AdaAttN [51]	27.44	52.96	0.071
Stytr2 [65]	23.72	61.63	0.103
MCCNet [24]	36.47	62.89	0.015
HSTN *σ* = 10	37.64	62.27	0.017
HSTN *σ* = 25	34.79	63.44	0.018
HSTN *σ* = 35	33.12	63.69	0.019
HSTN *σ* = 50	32.77	64.08	0.021
HSTN *σ* = 75	28.68	65.91	0.024

## 5 Limitations, challenges and future work

We would like to highlight a couple of limitations of the proposed method. The first is the limited qualitative study, i.e., 30 subjects. The subjective study is always quite challenging, and therefore, we would like to conduct a large-scale subjective analysis of the improved HSTN method with existing and recent works as a future prospect. The second limitation is that of the user control with respect to labeled regions. In this regard, we would like to explore the semantic segmentation aspect to provide more user control for stylizing an image. However, it would require the element of supervision for region decomposition. As the HSTN uses conditional probabilities to fuse multiple styles to a single content image, the use of stylized animation from a single image can be generated using the encoder-decoder block with post-processing techniques. The denoising block can also help in enhancing the stylized animation accordingly. The third limitation or we can say future avenue is to integrate the HSTN framework with immersive technologies such as Metaverse, spatial computing, non-fungible tokens, and Multimodal networks. We believe that such integration would not only enrich the HSTN framework with new techniques and methodologies but also provide a sense of realization to the users.

## 6 Conclusions

This paper proposes a novel network architecture, i.e., a Hierarchical Style Transfer Network (HSTN), that can generate visually compelling results with style control. The style control allows the user to style up or down the resultant image using the sigma value respectively. To achieve the task, we propose a fixpoint control loss in the encoder-decoder block and add the denoising element in the loss block of HSTN. We provide extensive experimental results and comparison with the existing methods such as optimization, single-style, multiple-style, and arbitrary style-based methods. It should also be noted that the proposed HSTN also liberates the use in choosing the level of stylization to be achieved. For instance, the user can only use two blocks for the stylization, i.e. HSTN-D or achieve multiple style transfers with HSTN-L. We also conducted a user evaluation for subjective analysis, revealing that the HSTN generates the most unique and visually appealing stylization results. In addition, we conducted experiments and comparative analysis with respect to the style and content classification scores. Results show that our method can compete with state-of-the-art methods while achieving a competitive trade-off between content and style classification scores, i.e., 37.64 and 62.27, respectively. This suggests that the proposed method can learn style representations effectively while preserving details from the content image. We also show that the proposed work is reasonably fast by reporting the inference times, which makes the proposed method suitable for integration in real-world applications. We also report the results for content and style classification scores while varying the sigma value, which justifies our claim for granting control to the user for image stylization.

## Funding

The authors are thankful to the 10.13039/100019725Deanship of Scientific Research and under the supervision of the Scientific Research Centre at 10.13039/501100005911Najran University for funding this work under the Research Centers Funding program grant code NU/RCP/SERC/12/4.

## CRediT authorship contribution statement

**Sunder Ali Khowaja:** Writing – original draft, Project administration, Investigation, Formal analysis, Data curation, Conceptualization. **Sultan Almakdi:** Writing – original draft, Project administration, Investigation, Formal analysis, Data curation, Conceptualization. **Muhammad Ali Memon:** Writing – original draft, Project administration, Investigation, Formal analysis, Data curation, Conceptualization. **Parus Khuwaja:** Writing – original draft, Project administration, Investigation, Formal analysis, Data curation, Conceptualization. **Adel Sulaiman:** Writing – review & editing, Visualization, Validation, Resources, Methodology. **Ali Alqahtani:** Writing – review & editing, Visualization, Validation, Resources, Methodology. **Asadullah Shaikh:** Writing – review & editing, Visualization, Validation, Resources, Methodology. **Abdullah Alghamdi:** Writing – review & editing, Visualization, Validation, Resources, Project administration, Methodology.

## Declaration of Competing Interest

The authors declare that they have no known competing financial interests or personal relationships that could have appeared to influence the work reported in this paper.

## Data Availability

Data is available on request due to privacy/ethical restrictions.
